# From purines to purinergic signalling: molecular functions and human diseases

**DOI:** 10.1038/s41392-021-00553-z

**Published:** 2021-04-28

**Authors:** Zhao Huang, Na Xie, Peter Illes, Francesco Di Virgilio, Henning Ulrich, Alexey Semyanov, Alexei Verkhratsky, Beata Sperlagh, Shu-Guang Yu, Canhua Huang, Yong Tang

**Affiliations:** 1 State Key Laboratory of Biotherapy and Cancer Center, West China Hospital, and West China School of Basic Medical Sciences & Forensic Medicine, Sichuan University, and Collaborative Innovation Center for Biotherapy, Chengdu, China; 2 International Collaborative Centre on Big Science Plan for Purinergic Signalling, Chengdu University of Traditional Chinese Medicine, Chengdu, China; 3 Rudolf-Boehm-Institut für Pharmakologie und Toxikologie, Universitaet Leipzig, Leipzig, Germany; 4 Department of Medical Sciences, University of Ferrara, Ferrara, Italy; 5 Department of Biochemistry, Institute of Chemistry, University of São Paulo, São Paulo, Brazil; 6 Shemyakin-Ovchinnikov Institute of Bioorganic Chemistry, Russian Academy of Sciences, Moscow, Russia; 7 Sechenov First Moscow State Medical University, Moscow, Russia; 8 Faculty of Biology, Medicine and Health, The University of Manchester, Manchester, UK; 9 Department of Pharmacology, Institute of Experimental Medicine, Hungarian Academy of Sciences, Budapest, Hungary; 10 Acupuncture and Chronobiology Key Laboratory of Sichuan Province, Chengdu, China; 11 School of Basic Medical Sciences, Chengdu University of Traditional Chinese Medicine, Chengdu, China

**Keywords:** Molecular medicine, Target identification

## Abstract

Purines and their derivatives, most notably adenosine and ATP, are the key molecules controlling intracellular energy homoeostasis and nucleotide synthesis. Besides, these purines support, as chemical messengers, purinergic transmission throughout tissues and species. Purines act as endogenous ligands that bind to and activate plasmalemmal purinoceptors, which mediate extracellular communication referred to as “purinergic signalling”. Purinergic signalling is cross-linked with other transmitter networks to coordinate numerous aspects of cell behaviour such as proliferation, differentiation, migration, apoptosis and other physiological processes critical for the proper function of organisms. Pathological deregulation of purinergic signalling contributes to various diseases including neurodegeneration, rheumatic immune diseases, inflammation, and cancer. Particularly, gout is one of the most prevalent purine-related disease caused by purine metabolism disorder and consequent hyperuricemia. Compelling evidence indicates that purinoceptors are potential therapeutic targets, with specific purinergic agonists and antagonists demonstrating prominent therapeutic potential. Furthermore, dietary and herbal interventions help to restore and balance purine metabolism, thus addressing the importance of a healthy lifestyle in the prevention and relief of human disorders. Profound understanding of molecular mechanisms of purinergic signalling provides new and exciting insights into the treatment of human diseases.

## Introduction

Purines and pyrimidines have long been recognised as fundamental elements of bioenergetics; while their role as chemical transmitter was suggested 90 years ago, when in 1929, Albert Szent-Gyorgyi and Alan Drury discovered that intravenous injection of adenine into guinea pig disturbed cardiac rhythm, indicating extracellular signalling function.^1^ Nearly two decades later, Wilhelm Feldberg and Catherine Hebb reported that adenosine triphosphate (ATP) stimulated the sympathetic ganglion in the cat.^2^ In 1959 Pamela Holton demonstrated that antidromic stimulation of sensory nerves in the rabbit led to the release of ATP.^3^ These findings suggested transmitter function of purines in the nervous system. In 1972, Geoffrey Burnstock proposed the purinergic neurotransmission hypothesis, which described ATP as the non-adrenergic and non-cholinergic neurotransmitter.^4^ This hypothesis failed to convince most researchers at first, but has been gradually accepted after molecular cloning of purinergic receptors which mediate signal transduction in response to extracellular purines has been accomplished. Purinergic receptors are commonly divided into two classes based on agonist selectivity, namely P1 adenosine receptors, and P2 nucleotide receptors (also known as ATP receptors).^5^ These receptors are further sub-classified into several subtypes, which are diffusely expressed in tissues and are activated by different purine derivatives, thereby exerting specific physiological functions.

**Fig. 1:**
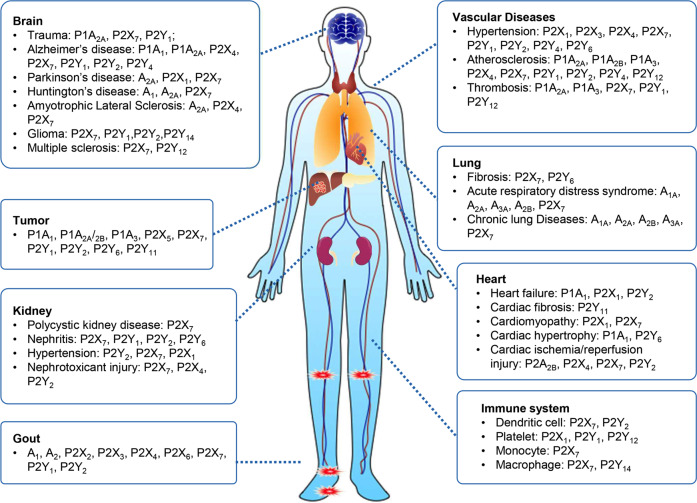
The causative role of purinergic signalling in human diseases. Purinoceptors, including P1, P2X and P2Y receptors, are diffusely expressed in every human body part, such as the nervous system, circulatory system, respiratory system, immune system, urinary system and others. Dysregulation of purinoceptors function leads to various diseases, including neurological, rheumatic, cardiovascular, cancer diseases and so on

Purinergic signalling interacts with other signal molecules to form a complex network, regulating numerous cellular processes including proliferation, differentiation, and death. For instance, ATP activates metabotropic P2Y receptor to promote cell proliferation, while activation of ionotropic P2X_7_ receptor arrests growth through recruiting protein kinases p38/MAPK and SAPK/JNK.^6^ Given the key role of purines in fundamental metabolic processes, and the involvement of purinergic transmission in the regulation of fundamental physiological processes (such as blood coagulation^7^ and neurotransmission^8^), deregulation and malfunction of the purine/purinergic system contributes to pathophysiology of numerous diseases, including gout, diabetes, neurological disease, osteoporosis and cancer (Fig. 1).

The best-known purine-related disease is gout, which results from accumulation of urate crystals in joints. Deposition of urate crystals is frequently associated with a purine-rich diet (i.e. seafood, animal offal, beer, etc.) or with renal insufficiency. To date, several drugs targeting purine metabolism or purinergic receptors have been developed (including tecadenoson, regrelor, AF-219, JNJ-54175446, PPADS, A317491, etc.), and many such drugs underwent successful trials or have been approved by the US Food and Drug Administration (FDA) (for example regadenoson, istradefylline, dipyridamole, clopidogrel, prasugrel, cangrelor, ticagrelor, etc.), further highlighting the importance of targeting purinergic signalling in the clinic. In this review, we shall briefly summarise main concepts and recent findings in purine biogenesis and metabolism, with a focus on the role of purinergic signalling in cellular functions and several human diseases including neurological disorders, rheumatic diseases and cancer. We shall also discuss therapeutic approaches, with a focus on traditional Chinese medicine (TCM) as a novel option for restoring the balance of purine metabolism.

## Purinosome as the fundamental unit to regulate purine metabolism

**Fig. 2:**
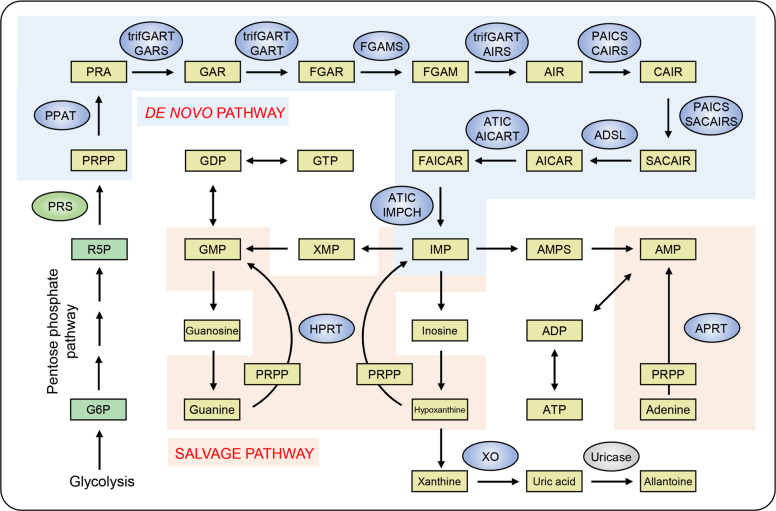
De novo and salvage pathway for purine synthesis. PRPP generated from glycolysis and pentose phosphate pathway (green) serves as substrate for de novo purine synthesis (blue background). After 10-step reaction catalysed by six enzymes with different functional domains, IMP is produced as the end of de novo pathway. Salvage pathway (orange background) is mainly catalysed by two enzymes, namely HPRT and APRT. In this pathway, PRPP is also needed, and several nucleosides serve as co-substrates for the production of nucleotides. The final metabolite uric acid is further oxidised by uricase (grey), which is expressed in most organisms but lost in humans and a part of primates

Purine metabolism affects a broad range of cellular processes, including energy production and DNA/RNA synthesis. ATP is hydrolysed to ADP and further to AMP, thereby meeting energy demand and facilitating nucleotide assembly. Cyclic AMP (cAMP) is the omnipresent second messenger controlling cellular physiological responses, further highlighting the crucial role of purine metabolism for the proper function of organisms. AMP can be dephosphorylated into adenosine, which is shuttled across cell membrane via equilibrative and concentrative nucleoside transporters (respectively, ENT and CNT)^9^. Adenosine translocation by ENT equalises adenosine levels on both sides of cell membrane, while CNT transports adenosine against a concentration gradient into the cells to maintain a high adenosine level inside the cell.^9^ Adenosine can be either recycled as AMP by phosphorylation, or converted into uric acid as the final metabolite. On the other hand, AMP and other purine nucleotides (i.e. inosine monophosphate (IMP), xanthosine monophosphate (XMP), and guanosine monophosphate (GMP)) can be converted into each other therefore generating a cellular purine pool. There are two major pathways maintaining this pool, namely the salvage cascade and de novo synthesis (Fig. 2).^10^ The salvage pathway provides purine nucleotide source by recycling degraded bases. In this cascade, AMP can be generated from adenine, this reaction being catalysed by the adenine phosphoribosyltransferase (APRT).^11^ Other purine nucleotides such as IMP and GMP can be produced from hypoxanthine and guanine, respectively; these processes are catalysed by hypoxanthine-guanine phosphoribosyltransferase (HPRT).^12^ Cells prefer salvage pathway over the de novo synthesis because of lower energy costs. Besides, many cellular populations (for example brain or bone marrow cells) lack the de novo purine synthesis cascade, being thus highly dependent on the salvage pathway.^13,14^

When purines are in deficit, the energy intensive de novo pathway is upregulated to meet increasing purine demand. Briefly, de novo purine synthesis pathway begins with the consumption of phosphoribosyl pyrophosphate (PRPP), and terminates with the production of IMP. The entire de novo pathway is comprised of 10 highly regulated reactions but only six enzymes are identified as direct participants, because some of enzymes are multi-functional thus capable of catalysing more than one reaction.^15^ For instance, trifunctional purine biosynthetic protein adenosine-3 (trifGART) catalyses three steps, namely conversion of 5-phosphoribosylamine (PRA) to glycinamide ribonucleotide (GAR), of GAR to N-formylglycinamide ribonucleotide (FGAR), and N-formylglycinamidine ribonucleotide (FGAM) to aminoimidazole ribonucleotide (AIR) by its GAR synthetase (GARS), GAR transformylase (GART), and AIR synthase (AIRS) domain, respectively.^16^ Two other enzymes, the multifunctional protein ADE2 (PAICS) and bifunctional purine biosynthesis protein PURH (ATIC), catalyse two steps.^17,18^ Remaining three enzymes, namely PRPP amidotransferase (PPAT), FGAM synthetase (FGAMS), and adenylosuccinate lyase (ADSL) are identified as monofunctional enzymes that catalyse only one reaction during the de novo purine synthesis.^19–21^ In addition to higher energy cost, the de novo pathway consumes more amino acids (i.e. glutamine, glycine, aspartate) and additional metabolites (i.e. formate and carbon dioxide) compared with the salvage pathway. This metabolic shift resembles several hallmarks of cancer: rapidly proliferating cancer cells require more amino acids (particularly glutamine) and favour acidic tumour microenvironment, suggesting a potential link between upregulated de novo purine synthesis and carcinogenesis. This matter will be discussed in following section.

To improve the utilisation of substrates and accelerate metabolic flux, metabolic enzymes tend to form complexes known as metabolon, frequently found in numerous metabolic pathways, including glycolysis and tricarboxylic acid cycle (TCA cycle). For example, the metabolon of glycolysis, termed a glycosome, is a multi-enzyme structure containing a series of glycosomal enzymes including hexokinase (HK), phosphofructokinase (PFK), alanine transferase (ALT) and many others.^22^ Similar metabolon also exists in the de novo purine synthesis pathway. Using fluorescence imaging technique, all six enzymes in the de novo pathway have been found to interact with each other to form a metabolon, defined as “purinosome”.^23^ In response to limited purine supplement, the purinosome promotes production of purines to meet nutrient demand. In HPRT1-mutated cells, that lack purine salvage capability, purinosome assembly undergo a 25% increase.^24^ In purinosome-rich cells, a 3-fold increase in IMP level was observed compared with normal cells.^25^ Binding efficiencies among six enzymes within purinosome are not comparable. The three enzymes PPAT, trifGART and FGAMS bind each other with a strong interaction thus forming the core of purinosome. In contrast the interaction of PAICS, ADSL and ATIC is relatively weak making them peripheral components.^26^ In addition to six de novo synthesis enzymes, some other purine-related enzymes are included in the purinosome, indicating that the purinosome is not only involved in the de novo purine synthesis, but contributes to other purine metabolic pathways. One of these additional components is adenylsuccinate synthase (ADSS), which catalyses the biogenesis of AMP from IMP.^25^ The inosine monophosphate dehydrogenase (IMPDH) that catalyses conversion of IMP to XMP, is also a member of purinosome.^25^ Several non-enzyme proteins that seem to be irrelevant for purine metabolism are also included in purinosome, such as the chaperones HSP70 and HSP90.^27^ The formation of purinosome is highly dynamic, for the depletion of purines in the medium promotes the assembly of purinosome, while a purine-rich medium leads to disassociation of the enzyme complex.^23^ These observations suggest that under purine-rich or depleted conditions, cells are capable to perceive extracellular purine level and regulate de novo purine synthesis by dynamically controlling the assembly and turnover of purinosome.

Although the dynamic regulation of purinosome formation and disassembly has been described long time ago, the underlying molecular cascades remain largely undefined. The finding that key components of purinosome undergo phosphorylation implies that certain kinase signalling pathways are critical to this process. A high-throughput study targeting human kinome revealed that the mammalian target of rapamycin (mTOR) facilitates the colocalisation of purinosome with mitochondria, which are required for optimal function of the purinosome itself.^28^ Given the high energy demand of the de novo purine synthesis, the spatial colocalisation with mitochondria provides high concentrations of ATP to support a series of purinosome-associated enzymatic reactions. Purines thus generated can be immediately utilised for the production of ATP. It is not surprising that microtubule participates in this spatial organisation of purinosome, for cytoskeleton largely affects the formation and disassociation of protein complexes and regulates their subcellular localisation. This hypothesis is supported by the finding that the purinosome is co-localised with microtubule, and disruption of the microtubule network impedes de novo purine synthesis.^29^ Microtubule depolymerisation by nocodazole treatment abrogates the co-localisation between purinosome and mitochondria, indicating that microtubule-directed transport contributes to purinosome–mitochondria co-localisation.^30^ The mTOR indeed regulates microtubule-dependent protein transport.^31^ This evidence suggests that mTOR–microtubule–purinosome–mitochondria axis plays a central role in the complex formation and spatial regulation of purinosome. Apart from mTOR, other kinases may also be involved in this process. For instance, inhibition of casein kinase II (CK2) has been shown to induce a disassociation of purinosome clusters and affect de novo purine biosynthesis in HeLa cells.^32^ Moreover, 3-phosphoinositide-dependent protein kinase 1 (PDK1) has been found to regulate the core assembly of purinosome through its cytoplasmic activity, but independent of its membrane-bound activity.^33^ It is worth mentioning that G-protein coupled receptor (GPCR) signalling, which regulates a great number of kinases such as PI3K, PKA, Akt, PKC, facilitates purinosome assembly, suggesting that additional kinases are probably involved.^34^

## Aberrant purine metabolism causes hyperuricemia and gout

**Fig. 3:**
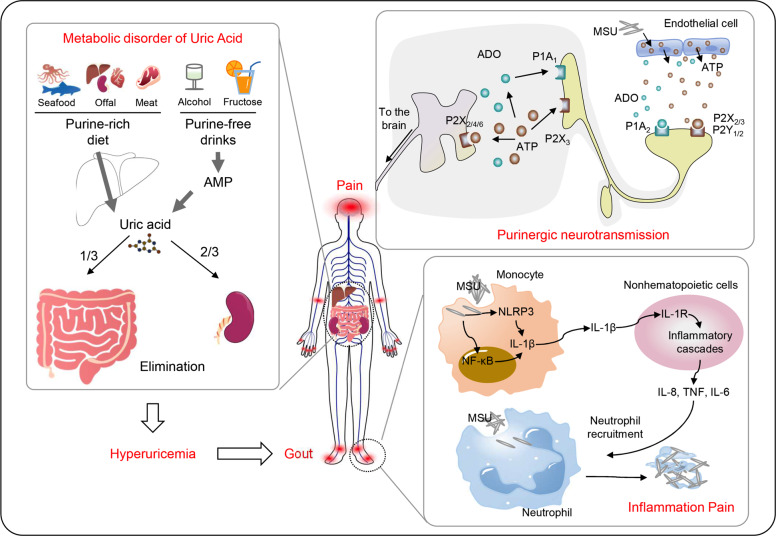
Dysregulated purine metabolism contributes to gout and pain. Purine-rich diet and several beverages, including meat, seafood, animal offal, beer and fructose containing drinks, lead to high level of uric acid in vessel, termed hyperuricemia. Deposition of urate in joint induces local inflammation. In this process, adenosine (ADO) and ATP function as neurotransmitters to activate P1 and P2 purinergic receptors, respectively. This purinergic neurotransmission induces intense pain in gout patients

Gout, the archetypal purine-related disease has an estimated incidence between 2.7% and 6.7% that is positively correlated with a western lifestyle.^35^ In mainland China, the incidence of gout was 0.9% in 2000–2005 but increased to 1.4% in 2011–2014, which reflects a rapid metamorphosis in the life style.^36^ Hyperuricemia is the major risk factor for gout, as it is caused by deposition of the monosodium form of urate (MSU) in the joints.^37^ Urate is the final metabolite of purine metabolism, and therefore dysregulation of purine catabolism contributes to gout (Fig. 3). There are multiple causes accounting for hyperuricemia and the deposition of MSU. Firstly, a purine-rich diet induces urate overproduction. Purine-rich foods include seafood, meat, animal offal, and alcoholic beverages, which all are associated with prosperous living condition. Alcohol and certain purine-free drinks, including fructose-containing beverage are also risk factors due to their ability to accelerate nucleotide breakdown, therefore increasing urate production.^38–40^ Conversion of fructose into fructose-1-phosphate requires consumption of ATP, leading to increased AMP level. Accumulated AMP enters into purine catabolic pathway to produce uric acid.^41^ Similarly, alcohol metabolism in liver consumes a large amount of ATP to generate AMP and thereby contributes to hyperuricemia.^42^ Secondly, loss of uricase (also known as urate oxidase) also probably leads to MSU deposition. In most organisms from bacteria to mammals, urate is oxidised by uricase to form soluble allantoin to avoid deposition. However, in humans and parting in some primates, this enzyme is lost.^43^ Given that urate also functions as an antioxidant, loss of uricase provides substantial advantages for humans against oxidative stress-related diseases such as neurodegeneration and cancer.^44^ From this perspective, potential side effects should be considered while treating gout with recombinant uricase.^45^ Thirdly, compromised excretion by kidneys and gut largely contributes to hyperuricemia. Approximately two-thirds of uric acid is excreted by kidneys, while gastrointestinal tract is responsible for the remaining one-third.^46^ This explains why the elders, in whom renal and gastrointestinal function is impaired, are more likely to have gout.

Mechanistic studies revealed that transporter-mediated urate traffic is closely associated with hyperuricemia induced by renal and gastrointestinal underexcretion. Serum urate level is mainly determined by four transporters, namely URAT1 (encoded by gene SLC22A12), GLUT9 (SLC2A9), NPT1 (SLC17A1) and ABCG2.^47^ In kidney, proximal tubular cells express all four transporters to balance serum urate. URAT1 localises at the apical side of proximal tubular cells, importing urate from renal tubule into lining cells.^48^ A fraction of cellular urate is then transported to blood via GLUT9, which localises at the basolateral side of proximal tubular cells.^49^ Alternatively, cellular urate can also be excreted into renal tubule via ABCG2 and NPT1, both of which localise at the apical side of proximal tubular cells.^50,51^ ABCG2 has been also found on the apical side of enterocyte, excreting urate into intestinal lumen.^52^ Clinical data suggest that a number of single-nucleotide polymorphisms (SNPs) exist in the encoding genes, leading to gain-of-function or loss-of-function in these four transporters. Thus, gain-of-function SNP in URAT1 and GLUT9, as well as loss-of-function SNP in ABCG2 and NPT1 increase the risk of gout. In contrast, loss-of-function mutations of the former two genes or gain-of-function mutation in the latter two may reduce the risk of gout. For instance, R90H and W258X non-functional mutations of URAT1 have been shown to decrease the risk of hyperuricemia.^53^ In addition, loss-of-function mutations of GLUT9 (i.e. C210F, N333S, etc.) are associated with lower serum urate.^54^ Introduction of the Q141K mutation (linked to a common SNP rs2231142) to ABCG2 halved urate transport hence reducing its contribution to development of gout.^55^ The gain-of-function mutation, I269T on NPT1, facilitates urate clearance and decreases the risk of gout.^51^ Moreover, these critical transporters might be regulated by genetic variants present in other genes. A recent genome-wide association study (GWAS) identified 183 loci associated with serum urate levels. Among these SNPs, the T139I on HNF4A upregulates its ability to activate ABCG2 promoter, leading to ABCG2 overexpression, which is negatively associated with serum urate level.^56^ Such genetic modifications are much more complicated, thus again highlighting the importance of a balanced diet in the prevention and relieve of hyperuricemia.

The definition of hyperuricemia is based on the laboratory data showing that urate begins to crystallise at a concentration of 0.41 mmol/L.^57^ Those crystals, known as MSU depositions, can be detected by microscopy and used as the gold standard for the diagnosis of gout. The preferred sites of MSU deposition are joints, including first metatarsophalangeal joint, ankle, knee, and organs such as the kidney. Deposition of MSU leads to acute inflammation and intense pain known as gout flare. Inflammation at affected joints is largely induced by the recruitment of immune cells (i.e. infiltrating macrophages, neutrophils, etc.) and accumulation of pro-inflammatory cytokines (i.e. IL-1β, IL-6, etc.).^58^ Mechanistic studies demonstrated that MSU crystals bind to membranes of macrophages instigating potassium efflux and the activation of NLRP3 inflammasome.^59^ This allows autocleavage of pro-caspase-1 into its mature form, which in turn processes pro-IL-1β to bioactive IL-1β. After activation of macrophages, neutrophils are recruited to inflammatory sites where they generate reactive oxygen species (ROS) and pro-inflammatory cytokines, thereby enhancing gout flare.^60^ Gout flare is a self-limiting process that resolves within about 10 days, probably because of build-up of anti-inflammatory cytokines including IL-10, TGFβ1 and IL-37.^61,62^ Neutrophils also play a role in the resolution of gout flare. Mounting a defensive response, neutrophils release their contents including chromosomal DNA and proteins to form neutrophil extracellular traps (NETs), which sequester pro-inflammatory cytokines such as IL-6 and TNF.^63^ Despite a transient relief of gout flare, MSU crystals fail to be removed from affected joint sites. Instead, MSU crystals bind to NETs, and aggregate in the connective tissue to form subcutaneous nodules termed tophi, the presence of which indicates a late stage of gout known as tophaceous gout.^64^ It may take decades to develop tophaceous gout after the first attack of gout flare, and a urate-lowering therapy is effective for arresting this severe disease.^65^ Tophaceous gout is frequently accompanied by bone damage. It has been shown that MSU crystals deposit on the synovial surface, causing a cortical break then progresses into bone erosion.^66^ Mechanistic studies demonstrated that MSU crystals reduce the viability of osteoblasts but enhance the development of osteoclast, leading to a resorptive state and subsequent bone loss.^67,68^ In line with it, a large-scale epidemiologic study revealed a 20% increased risk of osteoporosis in patients with gout compared with gout-free controls.^69^

Numerous studies indicate that lifelong urate-lowering therapy is the key for the management of gout. Based on metabolic pathways of urate production and turnover, several pharmacological strategies have been developed to achieve these blood urate targets. Uric acid is generated through the oxidation of xanthine by xanthine oxidase, therefore two inhibitors of xanthine oxidase, allopurinol and febuxostat, are currently used as the first-line defence agents.^70^ Allopurinol is a hypoxanthine analogue that binds and inhibits xanthine oxidase.^71^ In addition to decreasing uric acid production, inhibition of xanthine oxidase also results in an accumulation of hypoxanthine, which enters purine salvage pathway. This effect probably induces feedback inhibition of PPAT, the first enzyme of purine de novo synthesis to further block urate production.^72^ In clinical practices, dose escalation of allopurinol showed a better urate-lowering effect without increasing adverse responses.^73^ The safety of this drug is closely related to individual genetic and renal function. A rare but severe side effect, allopurinol hypersensitivity syndrome, may occur in patients carrying the HLA-B*5801 allele.^74^ Allopurinol usage can likely be a risk factor for renal disease.^75^ Allopurinol taken orally, rapidly metabolises into oxipurinol, which is slowly excreted by kidney.^76^ Given that gout patients frequently suffer from kidney impairment, administration of allopurinol probably increases the renal burden. This finding however, is controversial. A large-scale study indicated that allopurinol decreases the risk of chronic kidney diseases, suggesting the impact of allopurinol on kidney needs to be further examined.^77^ In contrast, another drug febuxostat is metabolised by liver and therefore it is not likely to impair kidney function. In hyperuricemic patients with chronic kidney diseases, febuxostat has been shown to be a better choice than allopurinol for delaying renal impairment.^78^ Febuxostat however, may cause acute liver injury.^79^ Moreover, treatment with febuxostat led to a higher mortality in gout patients with cardiovascular diseases, compared with allopurinol.^80^ Taken together, these observations suggest that both xanthine oxidase inhibitors hold curative effectiveness although potential side effects should not be overlooked. In addition to severity, therapeutic options depend on a variety of factors including individual genetic variation and the condition of kidney, liver as well as cardiovascular system.^35,81^

## Purinoceptors in pathophysiology

Purines exert essential pathophysiological effects by acting at purinergic receptors, the concept of which was introduced by Geoffrey Burnstock.^82^ Purinergic receptors are divided into two subfamilies known as P1 and P2 receptors. P1 receptors are G-protein-coupled receptors that recognise adenosine as endogenous ligand and are involved in a large number of physiological responses, such as modulation of heart rhythm.^83^ In early 1990s, four members of P1 receptor family A_1_, A_2A_, A_2B_ and A_3_, were cloned and characterised.^84^ These P1 receptor subtypes exhibit different affinity to adenosine, distinct pharmacology and tissue-specific expression patterns.^85^ Briefly, A_1_, A_2A_ and A_3_ receptors have high affinity, hence they are activated by physiological level (nM) of extracellular adenosine. On the contrary, the activation of A_2B_ requires much higher adenosine concentration (μM), which seems to be associated with pathological conditions such as hypoxia.^84,86^ A_1_ receptors are mostly localised in the nervous system, while the remaining three subtypes are diffusely expressed in a broad range of tissues including nervous system, spleen, colon, testis and others.^87^ Functionally, adenosine receptors are coupled with different G proteins, modulating the activity of adenylate cyclase (AC) in a positive or negative manner, thus affecting cytoplasmic cAMP. Both A_1_ and A_3_ receptors couple with G_i_ protein, hence their activation suppresses AC with subsequent decrease in cAMP level. In contrast, A_2A_ and A_2B_ are linked to G_s_ protein which stimulates AC.^85^ Extracellular adenosine activates A_1_ and A_2_ receptors of cardiac myocytes and cardiac fibroblasts, respectively, leading to a downregulated cAMP production in cardiac myocytes but an increase of cAMP level in cardiac fibroblasts, thereby preventing myocardial hypertrophy and fibrosis.^88^ Apart of AC, adenosine receptors regulate other effectors such as phospholipase C (PLC) and mitogen-activated protein kinase (MAPK).^89,90^ PLC controls the production of diacyl glycerol (DAG), an endogenous activator of protein kinase C, and inositol 1,4,5-trisphosphate (InsP_3_) that triggers Ca^2+^ release from endoplasmic reticulum (ER) Ca^2+^ store. Different adenosine receptors can form functional heteromeric complexes. For example, A_1_–A_2A_ complex binds to both G_i_ and G_s_ proteins thus triggering opposite cAMP signals. The outcome of this bidirectional complex depends on the adenosine concentration. Low adenosine concentrations preferentially activate the A_1_ subunit thus suppressing AC activity. When adenosine is high, A_2A_ component is activated causing increase in cAMP production.^91,92^ Although the pathophysiological function of P1 receptors is extremely complex, adenosine is generally considered a “protective” signal against stress conditions, because adenosine decreases energy intense activities and enhances nutrient support.^93^ This property makes P1 receptors attractive targets for treatments of cardiovascular and other diseases.^94^

The P2 nucleotide receptor family is divided into two subfamilies, P2X (ATP-gated ion channels) and P2Y (metabotropic GPCRs).^95^ P2Y receptors are activated by several nucleotides including ATP, UTP, ADP and UDP, whereas P2X are only activated by ATP. In 1993, the first two P2Y receptors were cloned, and the first two P2X receptors followed one year later.^96–99^ To date, seven P2X (P2X_1–7_) and eight P2Y receptor members (P2Y_1_, P2Y_2_, P2Y_4,_ P2Y_6_, P2Y_11_, P2Y_12_, P2Y_13_ and P2Y_14_) are recognised. By analogy to P1 adenosine receptors, P2Y receptors couple to specific G proteins thus differentially regulating downstream effectors including AC and PLC. P2Y_1_, P2Y_2_, P2Y_4_ and P2Y_6_ receptors couple to G_q_ protein, which activates PLC β to induce Ca^2+^ release from ER. In contrast, P2Y_12_, P2Y_13_ and P2Y_14_ couple to G_i_ protein, which inhibits AC to decrease cellular cAMP. P2Y_11_ receptors are linked to both G_q_ and G_s_ and their activation triggers cAMP and Ca^2+^ signalling.^100^ As mentioned above, P2Y receptors recognise not only ATP, but also other nucleotides as endogenous ligands, albeit with different efficacies. For instance, ADP is a more effective agonist at P2Y_1_ than ATP^101^. In contrast, P2Y_2_ has similar affinity for ATP and UTP, but is insensitive ER to ADP.^102^ Other P2Y receptors, including P2Y_4_, P2Y_6_, P2Y_11_, P2Y_12_, P2Y_13_ and P2Y_14_, prefer UTP, UDP, ATP, ADP, ADP and UDP-glucose, respectively.^103^ Additional chemical groups affect the potency of nucleotides at P2Y receptors. At P2Y_12_, 2-methylthio-ADP (2-MeSADP) is more potent than ADP, but adenosine-5′-O-(2-thiodiphosphate) (ADPβS) is less potent.^104^ Protein orthologs in different species have distinct preference and affinity for agonists. For example, at human P2Y_4_ UTP is the most potent agonist, whereas at rat P2Y_4_ UTP and ATP are equipotent.^105,106^

P2X receptors are archetypal ATP-gated cation channels,^107^ with ATP being the only known physiological agonist.^108^ The family of P2X receptors comprises seven members (P2X_1–7_) which assemble as homo- or heterotrimeric complexes. Among homotrimeric P2X receptors, P2X_1_ shows the highest affinity for ATP being activated at ATP concentrations <1 μM.^109^ The least sensitive member is the P2X_7_ receptor which requires ATP concentrations in hundred micromolar range for activation.^110^ High ATP sensitivity is frequently accompanied with a rapid desensitisation, whereas less sensitive subtypes might show persistent activation.^108^ According to ATP sensitivity and desensitisation time, homotrimeric P2X receptors are roughly classified into four subtypes. The first type includes P2X_1_ and P2X_3_ receptors, which are most sensitive to ATP and are rapidly desensitising. The second type includes P2X_2_ and P2X_5_ receptors with a lower ATP sensitivity and slower desensitisation. The P2X_4_ receptor belongs to a third type having similar ATP sensitivity, but prolonged desensitisation compared with the second type. The last type, represented by P2X_7_ receptors, shows the lowest ATP sensitivity and basically no desensitisation.^111^ The remaining member, the P2X_6_ receptor is not classified into these groups because it lacks the ability to form homotrimeric receptors.^112^

Similar to P2Y receptors, P2X receptors can be activated by ATP analogues with different potencies. For activation of P2X_1_, P2X_2_, P2X_3_ and P2X_5_, 2-MeSATP is equipotent with ATP. On the contrary, 2-MeSATP is 10-fold less potent than ATP at P2X_4_ receptors, and 100-fold less potent than ATP at P2X_7_ receptors.^113^ Composition of heterotrimeric channels may be rather complicated. For example, P2X_1/2_ heterotrimeric receptor is made of single P2X_1_ and two P2X_2_ subunits, whereas P2X_2/3_ receptor is assembled from a single P2X_2_ and two P2X_3_ subunits.^114,115^ The P2X_2/4/6_ receptor contains three different subunits, which is rarely observed for other P2X heterotrimers.^116^ This diversity of assembly confers to P2X receptors a large repertoire of physiological functions in different tissues.

Several P2 receptor agonists and antagonists have been developed for the treatment of human diseases. The examples are many; for instance, thienotetrahydropyridines that irreversibly inactivate P2Y_12_ receptors are used as antithrombotic drugs.^117^ The P2X_3_ receptor antagonist, AF-219 is under phase 2 clinic trail for treating refractory chronic cough.^118^ The rationale underlying these successful trails is that ATP generally acts as a proinflammatory signal, hence antagonists targeting P2 receptors may help restrain inflammation. To the contrary, adenosine restricts the inflammatory response, suggesting a therapeutic potential for P1 receptor agonists as anti-inflammatory drugs. Neladenoson, an A_1_ receptor agonist demonstrated positive results in the treatment of heart failure.^119^ Another promising application for modulators of purinergic signalling is a cancer therapy. Firstly, an inflammatory microenvironment has long been regarded as a hallmark of cancer.^120^ Secondly, tumour microenvironment is rich in ATP and adenosine.^121^ Thirdly, a variety of purinergic receptors are expressed in a wide range of tumour types.^122^ Moreover, ATP and adenosine are critical for the energy metabolism of cancer cells. Following scattered reports on the anti-cancer activity of some purinergic agonists,^123,124^ several clinical trials have been initiated to test the effectiveness of A_2A_ antagonists, alone or in combination with other established chemotherapeutic or immune check-point blockers, in cancer therapy.^125,126^ In addition, the discovery that the tumour microenvironment is rich in extracellular ATP^127^ has fuelled trials designed to exploit this peculiar trait of tumours to confer selectivity to anticancer drugs.^128^

## Purinergic neurotransmission in neurodevelopment and the pathogenesis of brain diseases

**Fig. 4:**
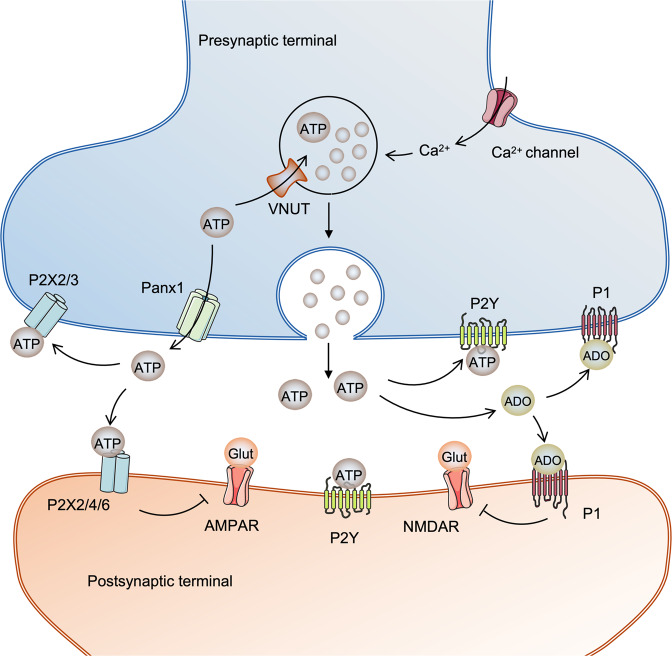
Purinoceptors mediates neurotransmission in nervus system. In presynaptic terminal, ATP is enriched in vesicles by transporter VNUT and then released in a Ca^2+^-dependent manner, or exported by other channels such as Panx1 independent of Ca^2+^. On one hand, extracellular ATP and its degradation product adenosine in turn activate purinoceptors in presynaptic terminal. This effect may regulate the release of other transmitters such as glutamate. On the other hand, extracellular ATP and adenosine can interact with postsynaptic purinoceptors, thereby regulating the excitability of neural cells. This event might also lead to the internalisation of AMPAR and NMDAR on postsynaptic membrane, leading to a decrease of glutamate-induced current. VNUT vesicular nucleotide transporter, Panx1 pannexin 1, AD adenosine, AMPAR AMPA receptor, NMDAR N-methyl-d-aspartate receptor

It is now well established that ATP is a bona fide neurotransmitter, contributing to the numerous functions of the nervous system (Fig. 4).^129^ In neuronal terminals ATP is accumulated within synaptic vesicles by vesicular nucleotide transporter VNUT/ SLC17A9.^130^ This transporter is widely expressed in the brain, and is central for ATP concentration (>100 μM) in the lumen of vesicles. Genetic deletion of VNUT suppresses ATP exocytosis in PC12 chromaffin cell line, suggesting VNUT-mediated vesicular storage of ATP is critical purinergic transmission.^130^ The ATP-rich vesicles are secreted by exocytosis in a Ca^2+^ regulated manner; treatment with Ca^2+^ chelators or exocytosis inhibitors abolishes ATP release.^131^ This Ca^2+^-dependent exocytosis of ATP vesicles can be directly visualised by live cell imaging.^132^ Several non-vesicular ATP release mechanisms were also reported, including large transmembrane channels represented by P2X_7_ receptors, connexins or pannexins.^133,134^ ATP is frequently released in combination with other transmitters. For instance, ATP has been found to be coreleased with noradrenaline (NA) and acetylcholine (ACh) in cortical synaptosomes^135^ and with glutamate in cortical slices^134^ ATP operating as a sole neurotransmitter has been found only in medial habenula.^136^

A fraction of released ATP activates P2 receptors in an autocrine/paracrine fashion. Remaining extracellular ATP undergoes hydrolysis to yield diphosphate nucleotides which activate several P2Y receptors, or is further broken down to produce adenosine thus activating P1 receptors. In this way, ATP or its metabolites stimulate para- or autocrinally purinergic receptors on the post- or presynaptic membrane, respectively. For example, ATP activates P2X_3_ and P2X_2/3_ receptors on the presynaptic membrane of spinal cord neurones, therefore regulating bladder micturition reflex.^137^ On the postsynaptic side, ATP-induced current can be evoked by the activation of P2X_2_, P2X_4_, P2X_6_ ionotropic as well as P2Y metabotropic receptors.^138^ As a cotransmitter, ATP may regulate other neurotransmission pathways. It has been shown that P2X_2_ activation induces internalisation of glutamate receptor (AMPAR) on postsynaptic side, leading to a decrease of synaptic current in hippocampal neurones.^139^ Another glutamate receptor, N-methyl-d-aspartate receptor (NMDAR), is also regulated by P2X receptors.^140^ In addition to the postsynaptic expression of glutamate receptors, the release of glutamate on the presynaptic membrane is regulated by both excitatory P2 receptors, including P2X_1_, P2X_2/3_, P2X_3_, and inhibitory receptors P2Y_1_, P2Y_2_ and P2Y_4_.^141^ The bulk of extracellular ATP is readily degraded by ectonucleotidases to adenosine, which activates P1 receptors and generally functions as an inhibitory signal. Among P1 receptors, A_1_ and A_2A_ are two major subtypes responsible for purinergic signalling in the brain.^142^ For instance, activation of A_1_ by adenosine results in a decrease of glutamate release and NMDAR expression on the presynaptic and postsynaptic membrane, respectively.^143–145^ Similarly, adenosine-mediated A_2A_ activation has been also found to inhibit glutamate release and NMDAR expression.^146,147^ Other details of purinergic neurotransmission have been extensively reviewed elsewhere.^148^

Purinergic signalling modulates embryonic neurodevelopment as well as neuroregeneration. Inner cell mass of blastocyst gives rise to the neural tube, in which progenitor cells differentiate to form peripheral and central nervous system, and these processes are largely regulated by purines and purinergic receptors.^149^ On rat embryonic day 11 (E11), P2X_3_ receptors are expressed in the hindbrain neural tube to support the development of autonomic nervous system.^150^ Genetic ablation of P2X_3_ receptors in mice eliminates inward currents in vagal sensory neurons and impairs their ability to sense gastric distension.^151^ Transcriptomics of the embryonic rat brain showed that P2Y_1_ receptors are hardly observed on E14 but are strongly expressed on E18, whereas P2Y_4_ maintains a comparable expression level throughout these days.^152^ In addition to this temporal regulation, expression of purinoceptors is region-dependent. On E9, P2X_5_ receptors are expressed throughout the spinal cord. However, 2 days later these receptors are detected only in the ventral horn, which is relevant to the development of motor fibres.^153^ Another purinergic receptor, P2Y_2_, is specifically expressed in the spinal motor nerves but not in the brain at E12.^152^ This purinergic receptor is co-localised with tyrosine receptor kinase A (TrkA) during neuronal differentiation, knockout of the latter abrogates the axonal growth of sciatic nerve in response to the stimulation of ATPγS.^154^ As to the P1 adenosine receptors, administration of P1 antagonists to pregnant mice delays migration of certain neurones, thus affecting brain maturation and leading to an impaired memory in foetal mice.^155^ Mechanistically, purinergic transmission modulates proliferation, differentiation, and migration of neural stem cells (NSCs) and neural progenitor cells (NPCs), contributing to neurodevelopment in foetal nervous system and neuroregeneration in the adult brain.^156^ Neurogenesis in the adult brain occurs in two niches, in subgranular zone (SGZ) in hippocampal dentate gyrus and subventricular zone (SVZ) in the lateral ventricle.^157^ The radial stem astrocytes which populate these regions symmetrically divide into daughter NSCs with self-renewal capabilities and asymmetrically into NPCs with highly proliferative properties; these latter give rise to neuroblasts. Several purinergic receptors regulate adult neurogenesis. For instance, the expression of P2X_7_ receptors has been detected in both embryonic SGZ/SVZ and in the adult hippocampus.^158^ In embryonic NSCs, activation of P2X_7_ receptors accelerates cell proliferation and inhibits differentiation.^159^ In the adult hippocampus, to the contrary, P2X_7_ receptors suppress the growth of NPCs and promote the differentiation into neurones and astrocytes.^160^ This effect may contribute to the damage repair in the adult brain by restricting inappropriate cell proliferation and facilitating neuronal production. Metabotropic P2Y_1_ receptors, stimulate proliferation and migration of neural progenitors, but do not affect their differentiation.^161^ In a P2Y_1_ receptor knockout mouse model, the downregulation of P2Y_1_ receptors was a prerequisite for the production of neuroprotective astrocytes during brain repair.^162^ In the presence of neurotrophins, ATPγS-mediated TrkA phosphorylation leads to neuronal differentiation in wild-type, but not in P2Y_2_ receptor-null mice, indicating that P2Y_2_ receptor is required for neurotrophin-induced regeneration.^154^ Elimination of unnecessary neural cells is a vital process during neurodevelopment, in which several purinoceptors including P2X_4_, P2X_7_, P2Y_2_ and P2Y_6_ receptors are involved.^163–166^ Modulation of neuronal apoptosis is largely attributed to excessive Ca^2+^ release from the ER, the process controlled by PLC/InsP_3_/InsP_3_ receptor axis downstream of purinoceptors.^167^

Aberrant purinergic transmission in the brain contributes to the pathogenesis of neurodegenerative disorders (i.e. Alzheimer’s disease, Parkinson’s disease, Huntington’s diseases, etc.) and neuropsychiatric diseases (i.e. depression, anxiety, addiction, etc.) (Table 1).^168–171^ Alzheimer’s disease (AD) is manifested by impaired cognition and memory and it is currently incurable. Deposition of β-amyloid aggregates is generally considered to be the primary cause of neuronal loss and a decreased level of ACh in brain, whereas other factors such as hyperphosphorylation of tau protein and disturbed metabolism of metal ion are also proposed. Numerous studies focusing on purinergic signalling provide another explanation for AD. As mentioned above, two P1 receptors A_1_ and A_2A_ are mostly responsible for the effects of adenosine in brain. Generally, the roles of A_1_ and A_2A_ in AD progression seem to be context-dependent, with both protective and deleterious consequences have been observed in the treatment of P1 agonists or antagonists. In AD patients, the expression of A_1_ receptors is downregulated in the hippocampus, whereas the expression of A_2A_ is upregulated in the periphery.^172,173^ Mechanistic study revealed that A_1_ receptors are co-localised with Aβ aggregates, and activation of A_1_ receptors leads to phosphorylation of PKC and ERK, which stimulates the production of soluble Aβ.^174^ In contrast, genetic ablation of A_2A_ receptors in mice protects against AD phenotypes, while optogenetic activation of this purinoceptor is sufficient to induce memory deficits.^175,176^ These findings suggest that A_1_ and A_2A_ may exert opposite functions during AD progression. Epidemiological studies indicate that caffeine, the non-selective antagonist of both A_1_ and A_2A_ receptors, effectively improves cognitive function and decreases the risk of AD.^177,178^ This observation implies that the deleterious effects of A_2A_ receptor may override the protective effects of A_1_ receptor during AD progression. Moreover, it has been reported that caffeine keeps blood–brain barrier intact, thus preventing β-amyloid deposition in brain.^179^ Given that the permeability of blood–brain barrier relies on multiple factors, the protective effect of caffeine might be attributed to other potential targets in addition to P1 receptors. Dysregulated ATP metabolism due to impaired mitochondrial function, for example, is one of the common characteristics in AD brain.^180^

**Table 1:** Several purinoceptors and their effects in neurotransmission in neurological diseases

Purinoceptors	Agonists	Antagonists	Distribution in nervous system	Regulated physiological functions	Related neurological diseases	References
A_1_	Adenosine, sleep deprivation	Caffeine	Hippocampus	Release of glutamate, expression of NMDAR	Alzheimer’s disease, depression	^143–145,172,173,191^
A_2A_	Adenosine	Caffeine, ZM241385	Basal ganglia	Release of glutamate, expression of NMDAR	Alzheimer’s disease, Parkinson’s disease, depression, pain disease	^143–145,172,173,189,190^
P2X_1_	ATP, 2-MeSATP, αβ-meATP	Suramin, PPADS, TNP-ATP	Spinal cord, cerebellum	Synaptic transmission	Parkinson’s disease	^141^
P2X_2_	ATP, 2-MeSATP	Suramin, PPADS	Spinal cord, hippocampus	Synaptic transmission, reflex of bladder micturition	Alzheimer’s disease, hearing loss	^137,139^
P2X_3_	ATP, 2-MeSATP, αβ-meATP	Suramin, A317491, PPADS, AF-219	Spinal cord, hindbrain, dorsal root ganglia	Reflex of bladder micturition, development of autonomic nervous system	Urinary incontinence, painful diabetic neuropathy, nociception	^137,150,151,207,208,212,213^
P2X_4_	ATP	5-BDBG, TNP-ATP	Hippocampus, hindbrain, spinal cord, cerebellum	Release of BDNF	Alzheimer’s disease, Pain disease	^215^
P2X_5_	ATP, 2-MeSATP	BBG, PPADS, Suramin	Spinal cord, ventral horn	Development of motor fibres	Not clear	^153^
P2X_7_	ATP, 2-MeSATP	Suramin, BBG, AZD9056	SGZ/SVZ, hippocampus, cerebellum	Proliferation and differentiation of neural progenitor cells, activity of α-secretase, release of ATP	Alzheimer’s disease, depression, pain disease	^134,135,158–160,185,186,214^
P2Y_1_	2-MeSADP, ADP, ATP	Suramin, PPADS	Hippocampus, cerebellum, spinal cord	Production of neuroprotective astrocytes	Alzheimer’s disease	^162^
P2Y_2_	UTP, ATP	Suramin	Spinal motor nerves	Neuronal differentiation and regeneration	Alzheimer’s disease	^154^
P2Y_4_	UTP, UDP	Suramin	Cerebellum, spinal cord	Not clear	Not clear	^141^
P2Y_6_	UTP, UDP	PPADS	Cerebellum, spinal cord, basal ganglia	Removal of unnecessary neural cells	Parkinson’s disease	^166^
P2Y_12_	2-MeSADP ADP	Ticlopidine, clopidogrel	Microglia	Neuroprotection	Pain disease	^215^

*2-MeSATP* 2-Methylthioadenosine-5′-O-triphosphate, αβ-meATP α,β-methyleneadenosine-5′-triphosphate, *TNP-ATP* 2′,3′-O-(2,4,6-Trinitrophenyl) adenosine 5′-triphosphate, *PPADS* pyridoxalphosphate-6-azophenyl-2′,4′-disulfonic acid, *BBG* Brilliant Blue G, *SGZ* subgranular zone, *SVZ* subventricular zone, *NMDAR* N-methyl-d-aspartate receptor, *BDNF* brain-derived neurotrophic factor

Several studies demonstrated that P2X_7_ receptor inhibition presents a neuroprotective effect in AD animal models.^181,182^ In rat hippocampus, β-amyloid aggregates have been shown to co-localise with P2X_7_ receptors.^183^ This co-localisation coincides with upregulated expression of P2X_7_ receptors, arguably linked to the production of reactive oxygen species (ROS).^184^ Mechanistically, P2X_7_ receptors promote β-amyloid deposition through α-secretase. Amyloid precursor protein (APP) is cleaved by three family members of secretase, namely α-, β-, and γ-secretase with different cleavage sites. Cleavage by α-secretase produces soluble fragment sAPP, which is harmless. Alternatively, β- and γ-secretase generate toxic β-amyloid which contributes to formation of senile plaques. Activation of P2X_7_ receptors was shown to inhibit the activity of α-secretase through activating glycogen synthase kinase 3 (GSK-3), leading to Aβ deposition in mice.^185^ However, opposite findings have been also reported demonstrating that P2X_7_ receptors activate α-secretase via modulation of MAPK pathway to produce sAPP.^186^ These contradictory results indicate that the role of P2X_7_ receptors in AD is yet to be characterised.

Depression is a neuropsychiatric disease characterised by decreased self-esteem and interest loss, with a low quality of personal life in aspects of sleeping, diet and general health. Epidemiological studies indicate that moderate coffee intake reduces the risk of depression, whereas excessive consumption may worsen the situation.^187,188^ This observation highlights possible involvement of A_1_ and A_2A_ adenosine receptors. Administration of ZM 241385, an inhibitor of A_2A_ receptor, decreases the immobility and isolation time in a rat depression model, indicating that activation of A_2A_ receptor contributes to depressive behaviour.^189^ Consistently, rats overexpressing A_2A_ receptor demonstrate depressive phenotypes.^190^ In contrast, stimulation of A_1_ receptor exhibits antidepressant effects. Activation of A_1_ receptors increases travel distance, whereas knockout of A_1_ receptors leads to immobility and other depressive behaviours in mice.^191^ Sleep deprivation, a widely used method to temporally relief depression, acts through activating A_1_ receptors.^192^ Consequently, moderate caffeine consumption predominantly inhibits A_2A_ receptors to with anti-depressant effect, whereas high dose of caffeine inhibits A_1_ receptors thus worsening depressive symptoms. This hypothesis is supported by the finding that antagonists for A_2A_, but not for A_1_ receptors, potentiate effects of classic antidepressants. Two antidepressant agents, agomelatine and tianeptine, were tested in combination with selective inhibitor of A_1_ receptors (DPCPX) and A_2A_ receptors (DMPX). Such combination revealed that co-treatment with DMPX, but not with DPCPX, increases the concentration of agomelatine and tianeptine in the brain thus potentiating their antidepressant effect.^193^ The P2 purinoceptors are also involved in depression. Psychological stress induces the release of ATP, activating P2X_7_ receptors thus leading to depressive behaviours in mice; the anxiety behaviour can be reversed by administration of P2X_7_ receptor antagonist.^194^ Genetic deletion of P2X_7_ receptor is anti-depressive in mice, and increases the efficacy of antidepressant drugs.^195^ Single nucleotide polymorphisms (SNPs) in A_2A_ and P2X_7_ receptor genes are linked to neuropsychiatric pathology. For example, the TT genotype at rs2298383 SNP in A_2A_ receptor predicts lower risk of depression compared to the CC or CT genotypes.^196^ The rs2230912 polymorphism also causes a Gln460Arg mutation in P2X_7_ receptor, which is associated with depression.^197^ These SNPs are believed to serve as potential indicators for early risk assessment of depressive disorders. Notably, several enzymes and metabolites of the purinergic transmission are also potential biomarkers for depression. It has been found that increased activities of adenosine deaminase (ADA) and xanthine oxidase (XO), as well as elevated level of xanthine and hypoxanthine correlate with a higher risk of depression.^198^ This evidence indicates that enzymes and metabolites involved in purine metabolism, together with purinoceptors, profoundly influence the pathophysiological function of nervous system.

## Purinergic transmission in mechanosensory transduction and pain

In 1977, the link between injection of ATP and other purines into human skin blisters and initiation of pain was discovered.^199^ Since then, a series of studies proved that activation of P2X receptors leads to pain-related defensive behaviours in animals.^200–202^ Acupuncture, an ancient medicinal codex developed in China over 4000 years, is used to relieve pain worldwide. Injection of A_1_ receptor agonist mimics the analgesic effect of acupuncture, suggesting it works through activating A_1_ receptors.^203–205^ To the contrary, P2 receptors commonly potentiate pain signals. The P2X_3_ receptor is the best characterised pain-related P2 receptor; which is predominantly expressed in small sensory neurones at dorsal root ganglia (DRG). Elimination of P2X_3_ receptors by toxin leads to a blunted response to acute pain in a rat model.^206^ In the context of human diseases, P2X_3_ receptor is also associated with painful diabetic neuropathy (PDN). Electroacupuncture decreases PKC-mediated upregulation of P2X_3_ receptors in DRG, which attenuates neuropathic pain and relieves PDN.^207,208^

Though positive effects have been observed in clinic, acupuncture and electroacupuncture are invasive procedures which do not fully satisfy the requirements of evidence-based medicine. Therefore, selective agonists (mostly for P1 receptors) and antagonists (mostly for P2 receptors) have been considered as promising analgesic agents.^93,209^ For example, the agonist of A_3_ receptors IB-MECA has significant pain-relieving effects in a mouse model.^210^ However, not all P1 receptor agonists with analgesic properties in animal proved their worth in clinical trials (the WAG 994 being an example.^211^ As to antagonists of P2 receptors, A-317491 a selective inhibitor of P2X_3_ and P2X_2/3_ receptors, is capable of relieving acute and chronic nociception in rat models,^212,213^ In addition, several P2 antagonists demonstrated efficacy in clinical trials. The P2X_7_ receptor antagonist AZD9056 has been shown to relief pain without obvious side effects in a phase II study.^214^ Similarly, antagonists for other P2 receptors including P2X_4_ and P2Y_12_ receptors demonstrated analgesic effects.^215^

## Purinergic transmission integrates immune system and modulates inflammatory responses

**Fig. 5:**
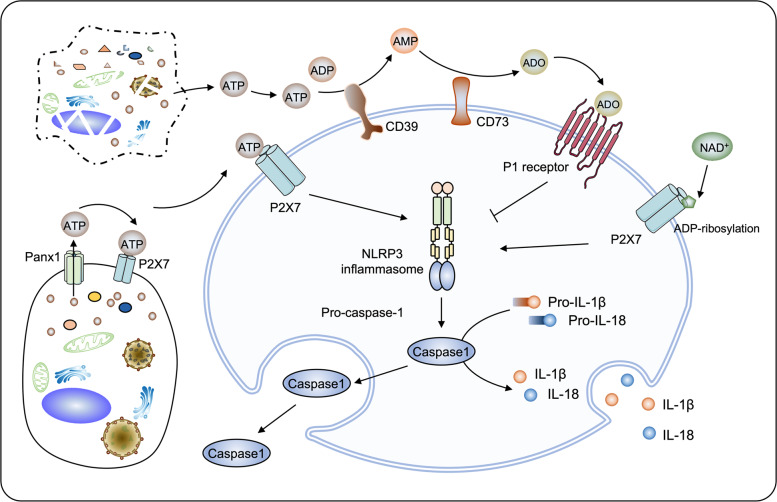
Purinergic signalling regulates immune and inflammatory responses. Besides necrotic cells, extracellular ATP can be released from living cells through Panx1 channel. Extracellular ATP activates P2X_7_ receptors, which subsequently activate NLRP3 inflammasome to induce the cleavage of pro-caspase-1. This effect leads to the maturation and release of caspase-1 thus initiating an immune or inflammatory response. Alternatively, extracellular ATP is ready to be degraded into adenosine (ADO) by ectoenzymes such as CD39 and CD73. The production of adenosine generally represses immune and inflammatory through binding with P1 receptors. In addition, P2X_7_ can be activated by ADP-ribosylation using NAD^+^ as an ADP-ribose donor

Gout is classified as a rheumatic immune disease, and pain is frequently companied by local inflammation.^216^ DNA, which is composed of purines, activates innate immunity when being translocated from nucleus or mitochondria into cytoplasm.^217^ This suggests a critical role for purinergic transmission in the regulation of immune system and inflammatory responses (Fig. 5). Purinergic receptors are expressed in nearly all kinds of immune cells. For instance, neutrophils express all four adenosine receptors and several P2 receptors including P2X_1_, P2X_7_, P2Y_2_ and P2Y_14_ receptors.^218^ In addition, several ectoenzymes such as CD39 and CD73 are expressed in immune cells; these enzymes regulate immune response through the production of adenosine from ATP.^219^ In general, ATP serves as a danger signal that activates immune cells, while adenosine attenuates inflammatory responses.^220^ Conceptually, extracellular ATP activates P2 receptors to initiate the immune response; subsequently ATP is converted to adenosine by CD39, CD73 and other ectoenzymes, leading to the activation of P1 receptors which contain inflammation. This model is supported by a large body of evidence. For example, CD39 and CD73 expressed in regulatory T cells (Tregs) convert extracellular ATP to adenosine, which suppresses the function of T effector cells.^205^ The recent finding that CD39 knockout mice possess enhanced immunity due to increased extracellular ATP and decreased adenosine, thus producing more CD8^+^ T in their spleen to resist bacterial infection further corroborates this model.^221^ Given the vital role of extracellular ATP in the onset of immune response, a long-standing question is that how ATP is released from the cells under infectious lesions or tissue-damaging conditions. First, ATP derives from necrotic cells.^222^ Second, ATP can be also released through several types of channels, such as pannexin 1 (Panx1).^223^ In contrast to necrosis, ATP released by Panx1 channel is regulated, for certain stimuli such as hypoxia induces Panx1 to form an ATP conduit, thereby accelerating ATP efflux.^224^

Functional P2X_7_ receptor is a trimeric complex with a variable conformation switches between open (ATP-bound) and closed states, which is one of the most intensively studied purinergic receptors that shapes immune responses.^225,226^ Mounting evidence suggests that P2X_7_ receptors control cytokine release and immune cell activation, thus modifying immune and inflammatory responses. For example, treatment with P2X_7_ receptor antagonist Brilliant Blue G (BBG) markedly decreases the level of plasma TNF-α in rats, thus suppressing the lipopolysaccharide (LPS)-induced febrile response.^227^ Inhibition of P2X_7_ receptors by periodate-oxidised ATP relieved allograft rejection in a cardiac transplantation mouse model; this relief being mediated by reduced T-cell activation.^228^ Stimulation of P2X_7_ receptors activates NLRP3 inflammasome, thereby regulating immune and inflammatory responses. Inhibition of P2X_7_ receptors prevents the activation of NLRP3, leading to a decreased caspase-1 cleavage and reduced sterile liver inflammation.^229^ Arguably there is a physical interaction between P2X_7_ receptors and NLRP3, which explains a molecular mechanism of P2X_7_ receptor-induced NLRP3 activation.^230^ Activated NLRP3 promotes the release of IL-1β through cleaved caspase-1, thereby leading to inflammation.^231^ In P2X_7_ receptor knockout mice, the release of IL-1β from peritoneal macrophages is largely blocked, indicating that P2X_7_ receptors are required for IL-1β secretion.^232^ The P2X_7_ receptor is the most potent activator of the NLRP3 inflammasome so far known, and therefore a most potent stimulator of mature IL-1β release. However, direct agonists of the NLRP3 inflammasome may trigger IL-1β release in a P2X_7_ receptor-independent fashion.^233^ Genetic deletion of P2X_7_ receptor in mice did reduce, but never completely abrogated the release of IL-1β.^234^ In addition to IL-1β, another cytokine released by NLRP3 is IL-18, and involvement of P2X_7_ receptor in IL-18 release has been documented.^235^

Other purines that accumulate at inflammatory sites, i.e. oxidised nicotinamide adenine dinucleotide (NAD^+^), may activate the P2X_7_ receptor synergistically with ATP, at least in the mouse.^236^ NAD^+^ provides an ADP-ribose that is covalently transferred to protein substrates, which reaction is catalysed by the ADP-ribosyltransferase (ART). This posttranslational modification, known as ADP-ribosylation, occurs at the extracellular domain of P2X_7_ receptor to initiate an inflammatory response.^237^ Mounting evidence suggests that NAD^+^ not only provide chemical group for ADP-ribosylation, but also mediate immune and inflammatory responses, at least partially, independent of the ADP-ribosylation. NAD^+^ shares considerable similarities with ATP in terms of immunity modulation. Firstly, NAD^+^ and ATP are both defined as danger signals.^238–240^ Secondly, both are released from necrotic or damaged cells.^241,242^ Thirdly, both extracellular NAD^+^ and ATP can accumulate at high concentration at the injury sites.^243,244^ Moreover, NAD^+^ itself is an agonist at P2Y_11_ receptor.^245^ This evidence indicates that NAD^+^ probably activates immune responses similarly to ATP. Indeed, intravenous injection of NAD^+^ significantly decreased the population of CD4^+^FoxP3^+^ Tregs, thereby promoting an antitumour response in vivo.^237^. On the contrary, inhibition of NAD synthetase NAMPT has been shown to decrease cytokine release, which can be used to attenuate acute intestinal inflammation.^246^

## Purinergic signalling regulates tumour development and progression

**Fig. 6:**
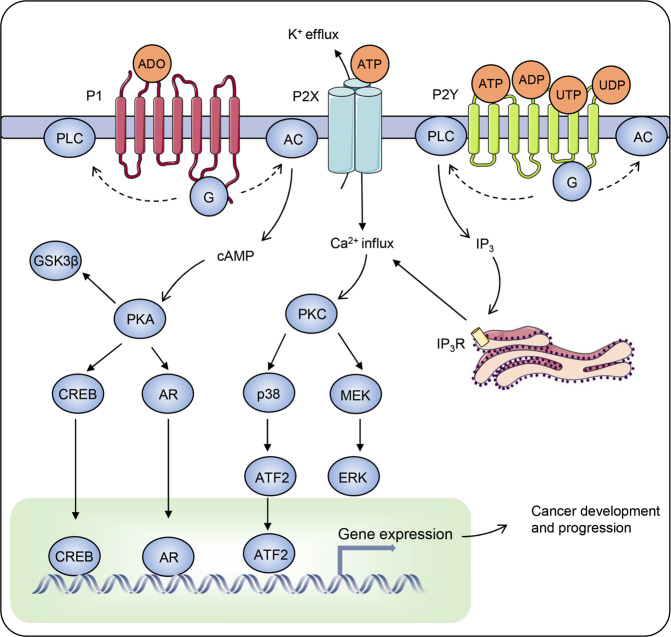
Purinergic signalling involved in the initiation and progression of cancer. Tumour microenvironment is rich in purines, including adenosine (AD) and other nucleotides. A panel of purinergic receptors are expressed in tumour cells to receive these extracellular purinergic signals. Activation of P1 and P2Y receptors leads to an altered activity of adenylate cyclase or phospholipase C (PLC) by coupling different G proteins (G), whereas activation of P2X receptors generates ion fluxes. These events change the level of several secondary messengers, such as cAMP, Ca^2+^ and InsP_3_. InsP_3_ binds to the InsP_3_ receptors on endoplasmic reticulum (ER), leading to the release of Ca^2+^ from ER. Thus, numerous proteins are regulated by these secondary messengers, including PKA, PKC, GSK3β, CREB, androgen receptor (AR), MEK, p38, ERK and ATF2. Among them are several transcription factors such as CREB, AR and ATF2, which induce a cancer-related transcription programme

Inflammatory and immunosuppressive microenvironment is a hallmark of cancer.^247^ Given the key roles of ATP and adenosine as pro-inflammatory and anti-inflammatory agents, respectively, this suggests a fundamental role of purinergic signalling in tumour development and progression (Fig. 6). Tumour microenvironment is rich in adenosine and ATP.^100,248^ It has been demonstrated that ATP accumulates at tumour interstitium at hundreds of micromolar while being nearly undetectable in the healthy samples.^249^ As discussed in the previous section, ATP controls immune cells, and accumulation of ATP triggers an antitumour immunity thus inhibiting tumourigenesis.^100^ Several types of P2 receptors are involved in the suppression of tumour growth, including P2X_5_, P2X_7_, P2Y_1_, P2Y_2_ and P2Y_11_ receptors.^250,251^ For example, P2X_5_ receptors contribute to the cytotoxic effects of T cells and suppression of chronic myeloid leukaemia.^252,253^ In contrast, knockout of P2X_7_ receptors decreases the population of CD8^+^ cytotoxic T cells.^254^ Therefore, ATP enrichment in tumour microenvironment seems to be an intrinsic antitumour mechanism induced by immune system. However, P2 receptors are found not only in immune cells, but also in cancer cells, making one wondering why tumour cells express “suicidal” molecules.^255^ This concern is even more pronounced due to the fact that extracellular ATP not only leaks from necrotic cells, but derives from active secretion associated with cancer cell metabolism.^256,257^ It appears that the antitumour immunity is potentiated at a high level of ATP (supplied pharmacologically), whereas a small increase of ATP (released endogenously) promotes proliferation of cancer cells.^258,259^ Indeed, overexpression or downregulation of P2X_7_ receptors has been shown to facilitate or suppress tumour growth, respectively.^260^ Apart from the growth-promoting function, low levels of ATP may also result in tumourigenesis through the release of immunosuppressive cytokines, such as IL-10.^261^ This evidence indicates that expression of certain P2 receptors provides advantages for tumours but also imply a pharmacologic approach towards cancer immunotherapy. According to this approach, ATP infusion has been shown to relieve the loss of weight and restore muscle strength in lung cancer patients.^262,263^

Adenosine similarly accumulates in tumour microenvironment and affects the development and progression of cancer. Extracellular adenosine largely derives from ATP breakdown, which is catalysed by ectoenzymes including CD39 and CD73. All four subtypes of P1 receptors are expressed in tumour cells. For instance, A_1_ receptors is overexpressed in renal cell carcinoma (RCC), and treatment with A_1_ receptors antagonist DPCPX inhibits RCC progression.^264^ Both A_2A_ and A_2B_ receptors highly expressed in neuroendocrine tumours promote proliferation of cancer cells.^265^ Activation of A_3_ receptors in glioblastoma increases the migration and invasion of cancer cells under hypoxia.^266^ As an immunosuppressive factor, adenosine supports tumour growth, at least partially. through repressing the antitumour immune responses. Genetic ablation or pharmacologic inhibition of A_2A_ receptors restores the antitumour activity of T cells, suggesting potential strategy for the cancer immunotherapy.^267,268^ Given that ectoenzymes are largely responsible for the production of adenosine in tumour microenvironment, suppression of these ectoenzymes (for example of CD73) has been shown to have considerable effects to enhance antitumour immune responses, and CD73-null mice are resistant to tumourigenesis.^269,270^ Adenosine seems to regulate cancer development in a dose-dependent manner in this resembling ATP. Endogenous adenosine converted from extracellular ATP promotes the migration of breast cancer cells, whereas exogenous adenosine or A_3_ receptors agonist IB-MECA suppresses their mobility.^271^

Purinergic receptors modulate the activity of downstream signalling cascades, thereby playing a role in cancer development and progression. Adenylate cyclase and PLC are two major effectors that couple with purinergic receptors and initiate downstream signallings. Alteration of cAMP was found in numerous malignancies.^272^ For instance, an increase in cAMP is associated with hepatocellular carcinoma (HCC).^273^ In contrast, the level of cAMP is decreased in lymphoma and ovarian cancer.^274,275^ Mechanistically, cAMP binds to the regulatory (inhibitory) subunits of protein kinase A (PKA), leading to its activation. The involvement of PKA in cancer has been extensively reviewed elsewhere.^276,277^ Another downstream effector, PLC, catalyses the production of diacyl glycerol (DAG) and InsP_3_ which both affect tumourigenesis.^278^ These secondary messengers play central roles in the signal transduction, linking purinergic signalling to other pathways especially those involved in embryonic and tumour development (i.e. Hippo, Wnt, Hedgehog, Notch and TGF-β).^279–283^ Genetic variations in the loci of purinergic receptors were demonstrated to have a prognostic value for tumour chemotherapy. Loss of P2X_7_ receptors allele promotes metastasis in anthracycline-treated breast cancer patients due to a deficient antitumour immunity compared with those patients harbouring a wild-type P2X_7_ gene.^284^

## Prevention and therapy of purine-related diseases by dietary and herbal interventions

Hyperuricemia induced by dysregulation of purine metabolism is associated with metabolic syndrome, which is also characterised by other features including obesity, hyperglycaemia, hypertension, hyperlipidemia and hyperinsulinemia. Similar to other metabolic syndrome manifestations, purine-related diseases are closely associated with the modern lifestyle.^285^ A 12-year perspective study suggested that intake of meat and seafoods increase the risk of gout to 1.41 and 1.51, respectively. In contrast, supplement with dairy products decreases the risk to 0.56.^286^ Although high abundance of purines in certain foods contributes to their propensity to induce gout, intake of purine-rich vegetables is not a risk factor.^287^ This difference might be attributed to the different bioavailability of purine derivatives from an animal or plant origin.^288^ Moreover, vegetables are beneficial for cardiovascular diseases and obesity, which are linked to aberrant purine metabolism.^289,290^ Therefore, vegetarian diet is beneficial regardless the purine content. Meat restriction calls for alternative protein sources, with legumes and dairy products are ideal choices. Inverse correlation between legume consumption and hyperuricemia were observed in independent cohorts.^291,292^ Dairy products have been found to reduce serum uric acid through the uricosuric effects of casein and lactalbumin, two proteins derived from the milk.^293,294^ Coffee is a beneficial beverage, being associated with a lower risk of gout.^295^ Caffeine has been shown to competitively inhibit xanthine oxidase, the enzyme producing uric acid from hypoxanthine and xanthine.^296^ However, caffeine alone failed to reduce the risk of gout, suggesting that other components of coffee contribute to its beneficial effect.^295^

**Fig. 7:**
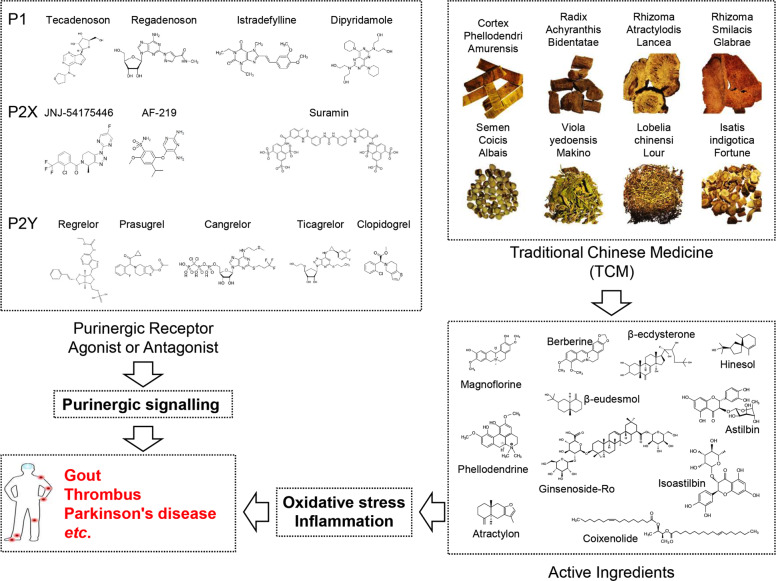
Traditional Chinese medicine and evidence-based medicine with therapeutic potential for purine-related diseases. Several TCM have been found to show considerable effect in the treatment of gout, including Cortex Phellodendri Amurensis, Radix Achyranthis Bidentatae, Rhizoma Atractylodis Lancea, Rhizoma Smilacis Glabrae, Semen Coicis Albais, Viola yedoensis Makino, Lobelia chinensi Lour, Isatis indigotica Fortune and so on. These TCM share similarities in terms of their components, which usually contain antioxidant and anti-inflammatory materials such as berberine, phellodendrine and magnoflorine. Combinational use of TCM as a formula might improve therapeutic efficacy and minimised adverse effect. In addition, FDA-approved drugs including istradefylline, dipyridamole, suramin, clopidogrel, prasugrel, cangrelor, ticagrelor, etc. are also therapeutic options targeting purine metabolism or purinoceptors

Dietary interventions are consistent with a theory of food homology according to traditional Chinese medicine (TCM), which describes that most foods hold therapeutic potential so that there is no absolute segregation between foods and drugs. In addition to physical methods such as acupuncture and cupping, TCM is mainly referred to herbal medicine that exhibits multi-targets effects. For instance, cortex phellodendri amurensis is one of the commonly used TCM for the treatment of gout.^297^ This effect might be attributed to its three components, berberine, phellodendrine and magnoflorine, all of which are capable of regulating inflammatory or antioxidant responses.^298–300^ Other TCM used for the treatment of gout include Radix Achyranthis Bidentatae, Rhizoma Atractylodis Lancea, Rhizoma Smilacis Glabrae, Semen Coicis Albais and Largehead Atractylodis Rhizome, with active ingredients such as β-ecdysterone, β-eudesmol, hinesol, atractylon and astilbin that exhibit anti-inflammatory, antioxidant or analgesic effects (Fig. 7).^301^ Importantly, in the clinic practice, different kinds of herbs are combined as complex formulae to minimise side effect and enhanced therapeutic outcome. For example, Erding granule (EDG) composed of Viola yedoensis Makino, Lobelia chinensi Lour, Isatis indigotica Fort and other components has been shown to reduce uric acid and suppress inflammation possibly via downregulating the expression of GLUT9 and URAT1 in a mouse model, suggesting this formula can be a potential agent for the treatment of hyperuricemia.^302^

It is worth mentioning that dietary and herbal interventions are useful for disease prevention or treatment at early stages. When diseases reach advanced stages, however, evidence-based medicine with specific drug target remains an optimal choice (Fig. 7). Hitherto, a number of drugs targeting purine metabolism or purinergic signalling have been developed. The US FDA database, contains several approved purinergic signalling target-related drugs, which include ADO injection [P1 receptor agonist used for the treatment of paroxysmal supraventricular tachycardia (PSVT) including that associated with accessory bypass tracts (Wolff–Parkinson–White syndrome)]; Cafcit (Caffeine citrate) injection (P1 receptor antagonist for the treatment of apnoea of prematurity); Lexiscan (regadenoson) injection (A2_A_ receptor agonist, used as a pharmacologic stress agent in radionuclide myocardial perfusion imaging, a test that detects and characterises coronary artery disease, in patients unable to undergo adequate exercise stress); Nourianz (Istradefylline) tablets (A2_A_ receptor antagonist used as an add-on treatment to levodopa/carbidopa in adult patients with Parkinson’s disease experiencing “off” episodes); Brilinta (ticagrelor) tablets (P2Y_12_ receptor antagonist, treatment for acute coronary syndrome, coronary artery disease, cardiovascular risk reduction, ischaemic stroke, and prophylaxis); Effient (Prasugrel) tablets (P2Y_12_ receptor antagonist, oral antiplatelet agent for the treatment of patients with acute coronary syndrome who are managed with percutaneous coronary intervention including coronary stenting); Kengreal (Cangrelor) injection (P2Y_12_ receptor antagonist, intravenous P2Y_12_ platelet inhibitor indicated for use in patients undergoing percutaneous coronary intervention to reduce the risk of periprocedural thrombotic events); Plavix (Clopidogrel bisulfate) tablets (P2Y_12_ receptor antagonist, indicated for the reduction of atherothrombotic events such as recent myocardial infarction, recent stroke, or established peripheral arterial disease, and acute coronary syndrome); and dipyridamole tablets (inhibiting the uptake of adenosine into platelets, endothelial cells and erythrocytes in vitro and in vivo, used as an adjunct to coumarin anticoagulants in the prevention of postoperative thromboembolic complications of cardiac valve replacement). In addition, a variety of drugs have been studied and applied in clinic for the treatment of different diseases although they have not got approval from FDA. For example, Diquafosol (tradename Diquas) is a P2Y_2_ receptor agonist that has been approved for the treatment of dry eye disease in Japan in 2010 and it is available for dry eye treatment in Japan, South Korea, Thailand, Vietnam, and China. Topical instillation of 3% diquafosol ophthalmic solution increased lipid layer thickness within 60 min in normal human eyes regardless of age^303^ and the recent real-world study showed that diquafosol 3.0% ophthalmic solution was well tolerated and effective in the long-term treatment (12 months) of dry eye.^304^ The Phase 3 trial for Denufosol (inhaled P2Y_2_ agonists for the treatment of cystic fibrosis) is in the progress. The Phase 2 clinical trial of AZD9056 (P2X_7_ receptor antagonist) demonstrated statistically significant decrease in Crohn’s Disease Activity Index from baseline in Crohn’s patients receiving 200 mg AZD9056 (UID for 4 weeks). Another P2X_7_ antagonist JNJ-54175446 is an orally bioavailable, central nervous system-penetrating; it is currently in Phase 2 clinical trial for the treatment of major depressive disorder (clinicaltrials.gov,NCT04116606). AF-219/gefapixant, a P2X_3_ receptor antagonist has been through a randomised, double-blind, placebo-controlled phase 2 study for the treatment of the refractory chronic cough^305^ and a randomised, double-blind, controlled, parallel-group, phase 2b trial for the treatment of refractory or unexplained chronic cough.^306^ All of these drugs should be strongly recommended as new therapies with widened clinic applications.

## Concluding remarks

Apart from serving as building blocks and energy source, purines regulate numerous cellular processes by initiating purinergic signalling. Purine metabolism regulated by purinosome determines the availability of purines, which act as endogenous ligands for a panel of purinergic receptors. Activation of purinergic receptors controls the production of various secondary messengers including cAMP, DAG, IP_3_ and Ca^2+^, thus leading to physiological consequences in normal conditions. In contrast, unhealthy diet, genetic variation or other pathological events disturbs purine metabolism, resulting neurodegeneration, gout, pain, inflammation and cancer. In addition to these disorders, purinergic signalling is related with many other diseases including cardiac, gastrointestinal, muscular, reproductive disorders and so on. Due to the extreme complexity of human body, we did not cover all these aspects, while we focused on representative examples.

As the novel drug target, purinergic signalling has a promising future in the development of new drugs or new indications due to current success in ADO, A_1_, A2a, and P2Y_12_ receptors. However, any of the four main purines, ATP, ADP, AMP, and ADO in the purinergic system, any of the three key enzymes, CD39, E-NPP, and CD73, and any of the 19 purinergic receptors, four P1 receptors (A_1_, A_2A_, A_2B_, A_3_), seven P2X (P2X_1–7_), and eight P2Y (P2Y_1,2,4,6,11–14_) need more attention in the future. In particular, the following therapies may come true in the near future: ATP for depression, AD; ADO for sleep disorders; CD73 for cancer or inflammation; A1 receptor for pain and sleep disorders; A2_A_ receptor for AD and PD; P2X_1_ receptor for bladder disorder and hypertension; P2X_3_ receptor for cough and hypertension; P2X_7_ receptor for cytokine release syndrome, inflammatory disease, brain disorders, and cancer; P2Y_2_ receptor for dry eye; P2Y_12_ receptor for pain and inflammation beyond current indications approved by FDA.

Though remarkable progress has been made in this field, several details remain to be elucidated for an in-depth understating towards purinergic signalling. For instance, how does each component of purinosome is well organised to form together and translocated to mitochondria? How to target a specific purinergic receptor without affecting other family members in the same subgroup? How to minimise potential side effects when targeting purinergic receptors due to multiple secondary messengers are simultaneously affected? These concerns need to be addressed before purine-based therapeutic strategies can be broadly applied for the treatment of relevant human diseases.^307–309^

We miss the creator of Purinergic Signalling^310,311^ Prof. Geoffrey Burnstock and we are very grateful for his great contributions to the discovery and characterisation of this novel molecular target for the development of promising drugs to treat human disease.
